# Factors associated with caregiving self-efficacy among primary informal caregivers of persons with dementia in Singapore

**DOI:** 10.1186/s12877-020-01951-8

**Published:** 2021-01-06

**Authors:** Gregory Tee Hng Tan, Qi Yuan, Fiona Devi, Peizhi Wang, Li Ling Ng, Richard Goveas, Siow Ann Chong, Mythily Subramaniam

**Affiliations:** 1 Research Division, Institute of Mental Health, Singapore, Singapore; 2 Department of Psychological Medicine, Changi General Hospital, Singapore, Singapore; 3 Department of Geriatric Psychiatry, Institute of Mental Health, Singapore, Singapore

**Keywords:** Informal caregivers, Dementia caregiving, Caregiving self-efficacy, RSCSE

## Abstract

**Background:**

Informal caregivers of persons with dementia (PWD) are often associated with negative health outcomes. Self-efficacy in dementia caregiving has been reported to have protective effects on caregiver’s health. This study aims to examine the factors associated with the domains of caregiving self-efficacy among informal caregivers in Singapore, a country with a rapidly aging population and a 10% prevalence of dementia among older adults.

**Methods:**

Two hundred eighty-two informal caregivers were recruited and data including participant’s caregiving self-efficacy, sociodemographic information, perceived social support, positive aspects of caregiving, knowledge of dementia, as well as behavioral and memory problems of care recipients were collected. A confirmatory factor analysis (CFA) was performed for the 3-factor model of the Revised Scale for Caregiving Self-Efficacy (RSCSE), and multiple linear regressions were conducted using the RSCSE subscales as dependent variables.

**Results:**

Our CFA found that the RSCSE 3-factor model proposed by the original scale developer was an acceptable fit among informal caregivers in Singapore. Having established that the 3-factor model of the RSCSE was compatible among our sample, a series of multiple regressions were conducted using each of the factors as a dependent variable. Regressions revealed several factors that were significantly associated with caregiving self-efficacy. Importantly, outlook on life was positively associated to all 3 domains of the RSCSE, while social support was positively associated with self-efficacy in obtaining respite and controlling upsetting thoughts.

**Conclusion:**

The 3-factor model of the RSCSE was found to be an appropriate fit for our sample. Findings from this study elucidated important novel insights into the factors that influences caregiving self-efficacy amongst informal caregivers in Singapore. Crucially, caregivers’ outlook on life and social support should be improved in order to enhance their caregiving self-efficacy.

## Background

The majority of persons with dementia (PWD) are being taken care of by family members or informal caregivers. These caregivers are often referred to as the invisible second patients [1], due to the negative physical and mental health consequences associated with being a caregiver to a PWD. Some risks that have been observed among dementia caregivers include having a compromised immune system [2, 3], increased odds of mortality [4], higher likelihood of contracting cardiovascular diseases [5], and being at a greater risk of developing depression and anxiety [6, 7]. Caregivers of PWD are also more likely to be smokers and obese [8], and susceptible to experiencing high burden and burnout [9, 10].

In this regard, it is worth highlighting that self-efficacy has been reported to have a protective effect on caregiver’s health. Self-efficacy is a construct that refers to an individual’s belief in performing confidently and capably in specific situations [11]. Conceivably, caregivers who possess a higher sense of efficacy are more likely to frame caregiving tasks as challenges that can be overcome, are better able to recover from setbacks, and are more likely to persist in finding ways to cope. On the contrary, caregivers with lower self-efficacy are more prone to ruminating on the consequences of failures and their personal deficiencies [11, 12]. Other studies documenting the impact of self-efficacy on caregiver of PWD have reported that self-efficacy for symptom management may be useful in alleviating the caregiver’s burden and depression [13, 14]. Additionally, caregiving self-efficacy has been reported to be inversely related to depressive symptoms [15], and to confer a protective effect against risks of cardiovascular disease [16].

Singapore is a country with a rapidly aging population and a 10% prevalence of dementia among older adults above the age of 60 years [17]. It is estimated that a third of the population in Singapore would comprise adults above 60 years of age by 2030 [18]. However, there is currently no insurance coverage for dementia in Singapore [19], and an estimate by Bupa and Alzheimer’s Disease International puts the number of PWD living with family in Singapore at 90% [20]. Moreover, a qualitative study in Singapore found that caregivers of PWD felt that there was a dearth of dementia-specific residential care centers [21]. Such a trend entails that many more individuals would have to take on the role of an informal caregiver to PWD, as it is unlikely for services such as dementia nursing homes to be commensurate with the demands. Given the protective effects that self-efficacy confers on the caregiver, it is therefore of great importance to understand the impact of caregivers’ sociodemographic characteristics and other caregiving related factors (knowledge of dementia, positive aspects of caregiving and social support), so as to better inform the design of more effective interventions that can improve a caregivers’ self-efficacy.

To our knowledge, only two studies [22, 23] till date have examined caregiving self-efficacy amongst dementia caregivers in Singapore. However, Tew et al. (2010) study only examined the effect of self-efficacy on caregivers’ decision to institutionalize the person with dementia but not the sociodemographic correlates of self-efficacy, nor other variables such as social support and knowledge of dementia. Although Tay et al. (2016) study did examine the relationship of self-efficacy with demographic variables; the study had a relatively small sample size (*n* = 84). Furthermore, Tay et al. (2016) used a generic caregiving scale with a unidimensional score, instead of a self-efficacy scale with multiple domains that is specific to dementia caregiving, such as the Revised Scale for Caregiving Self-Efficacy (RSCSE) [12]. Ideally, self-efficacy should be studied using a multi-dimensional approach. Bandura (2006) recommended that self-efficacy scales should measure specific functional domains which contain behaviorally detailed items, as self-efficacy is derived from how an individual appraises the experience of his or her own action in specific circumstances. In which case, domain-specific beliefs may better reflect a caregiver’s emotional states and challenges perceived towards various aspects of caregiving than if self-efficacy was measured as a global sense of mastery [24].

This study aims to investigate the dementia caregiving self-efficacy among a sample of informal caregivers in Singapore using the multi-dimensional RSCSE, which is a validated scale that has been used in many studies across various countries [25]. The primary aim of this study is to explore the significant correlates of caregiving self-efficacy among local dementia caregivers as such information will be informative for designing effective interventions. Secondarily, we also conducted a factor analysis to ensure internal reliability of the RSCSE assessment tool in the local context.

## Methods

### Participants and procedures

Using a convenience sampling strategy, a total of 282 participants were recruited between January 2017 to December 2018. A total of 433 potential participants were approached, 282 participants agreed to participate, and the response rate was approximately 65%. Participants were recruited at two sites, namely at the outpatient clinic of Institute of Mental Health (the only tertiary mental health institute in Singapore) and its satellite clinics, as well as from a psychogeriatric clinic in Changi General Hospital. Participants recruited had to be: (1) a Singaporean or Permanent Resident of Singapore who is at least 21 years of age; (2) the primary informal caregiver to an individual formally diagnosed with dementia; (3) literate in either English, Chinese, or the Malay language. Written informed consents were obtained from all participants. This study was approved by the Domain Specific Review Board of National Healthcare Group in Singapore and the relevant institutional ethics committee. More details about the study can be found in earlier articles [26–28].

### Study questionnaires

Trained interviewers administered a series of questionnaires to the participants to collect their sociodemographic information and the functional status of their caregiving recipient, as well as to assess their perceived self-efficacy in their caregiving experiences. The sociodemographic questionnaire consisted of items that asked caregivers about their age, sex, ethnicity, marital status, highest education qualification attained, employment status, relationship to care recipient, whether they were living with the care recipient, average hours of caregiving per week, and whether they had a helper at home.

The functional status of PWD was collected using the Activities of Daily Living Scale (ADL) [29] and the Instrumental Activities of Daily Living Scale (IADL) [30], both of which had been validated in Singapore [31, 32]. The ADL comprised 6 items pertaining to the care recipient’s disability in six basic self-care activities such as bathing, dressing, toileting, transfer, continence and feeding. The IADL consisted of 8 items, that measured eight instrumental related self-care activities (i.e., ability to use a telephone, shopping, food preparation, housekeeping, laundry, mode of transportation, responsibility for own medication, and ability to handle finances). During the interview, caregivers reported the level of assistance that the care recipient required to carry out these activities. The self-care impairment scores for the care recipient were derived by totaling up the number of ADL and IADL. Additionally, care recipient’s memory and behavioral problems were also measured, using the Revised Memory and Behavioural Problems Checklist (RMBPC) [33]. Modelling on a previous study in Singapore [34], only 15 items from the RMBPC were used in this current study, and caregivers were asked to report whether the care recipient displayed any of the problems described by the 15 items in the week prior to time of recruitment.

Other caregiving related variable that were collected in this study included: 1) perceived social support measured using the 8-item scale by Pearlin et al. [35]; 2) positive aspects of caregiving, measured by the 9-item Positive Aspects of Caregiving Scale (PAC) [36]; and 3) knowledge of dementia using the Dementia Knowledge Assessment Scale (DKAS) [37]. Scores for social support were derived by summing up participants’ response to all 8 items on the scale to form a continuous variable, with higher scores indicating greater social support. For positive aspects of caregiving, participants’ responses were collated into 2 factors for analysis, namely Self Affirmation and Outlook on Life [36, 38]. Participant’s knowledge of dementia was analyzed on domain-specific level, using the 3-factor model structure that was proposed in an earlier work [28].

Caregiver’s levels of self-efficacy in caregiving was assessed with the RSCSE, and participants were read a series of 15 items describing issues or scenarios that they may encounter pertaining to obtaining respite, dealing with care recipient’s memory and behavioral problems, as well as negative thoughts about their role as a caregiver [12]. Caregivers were asked to rate their confidence at overcoming these difficulties, ranging from 0 to 100, or not applicable if they felt that the described item is not relevant to their present caregiving experience. The RSCSE has a 3-factor model, namely self-efficacy in obtaining respite (SE-OR), responding to disruptive behaviors (SE-RDB) and controlling upsetting thoughts (SE-CUT), that individually consist of five items. Factor scores were tabulated by the average summed scores of the five items, with higher score symbolizing better self-efficacy, omitting items with not applicable response from the calculation.

Permission for use of the DKAS and RSCSE were sought from the respective scale developers.

### Questionnaires translation

Many of the older adults in Singapore, especially those of Chinese and Malay ethnicity, are more fluent in their native language than in English which is the official language in Singapore. Hence, to ensure that participants are interviewed in their preferred language, the study team translated the English version of the questionnaires following a standard ‘translation back-translation’ procedure [39].

The translation process started with a single forward translation of the questionnaires by qualified bilingual individuals. A panel consisting of professionals and researchers then reviewed the translated questionnaires. Issues such as inadequate expressions in the translation, and discrepancies between translated and original version were highlighted by the panel members during this reviewing process. Subsequently, the panel and study team members discussed on ways to resolve those identified issues, followed by a back-translation of the questionnaires (by another researcher not involved in the process) so as to minimize the discrepancies. Pre-testing of the translated questionnaires were conducted with some potential participants who preferred to have the survey administered in the translated language over English. During this process, words or phrases in the translated scale which testers felt were ambiguous or perhaps incomprehensible in the local context were reflected to the study team. Based on the feedback gathered, necessary edits were made to achieve better conceptual equivalency, which gave rise to the final version of the translated questionnaires.

### Analysis

Descriptive analysis of the sample were performed using SPSS version 23. A confirmatory factor analysis of the RSCSE was carried out using the lavaan package under ‘R’ software. According to the scale developer, ‘not applicable’ is also a valid answer of the RSCSE. For this reason, even though missing value was used to represent this answer category during our data entry, missing values were still taken into the factor analysis. In order to best fit this condition, ‘pairwise deletion’ was used as the strategy to deal with missing data (i.e. ‘not applicable’ in this study). In the current study, an acceptable model was defined as 1) the comparative fit index (CFI) > 0.90; 2), the Tucker-Lewis index (TLI) > 0.90, and 3) the root mean square error of approximation (RMSEA) < 0.08 [40]. Subsequently, after deriving a suitable factor structure of the RSCSE among our sample, a few series of multiple regressions were conducted using SPSS with each of the RSCSE factors designated as dependent variable, while sociodemographic, PWD’s functional status (Summed scores of ADL and IADL, and total score of RMBPS) and caregiving related factors (perceived social support, Self Affrimation and Outlook on Life and DKAS factors) were all set as independent variables.

## Results

The mean age of participants was 55.6 ± 11.8, and average weekly caregiving hours were 54.9 ± 52.9. Majority of participants were Chinese (83.0%), female (75.2%), ever married (72%) and living with the care recipient (70.2%). Approximately half of all participants were employed (57.1%), with highest education level being N/O level (Secondary’s schools General Cert of Education) and below (42.6%), were daughters to the care recipient (55.3%) and had a domestic helper (57.1%). Refer to Table 1 for more details on the descriptive analysis of our sample.

**Table 1:** Sociodemographic Characteristics of Participants (*n* = 282)

	n	%
Sex		
Male	70	24.8
Female	212	75.2
Ethnicity		
Indian and Others	19	6.7
Malay	29	10.3
Chinese	234	83.0
Education		
N/O level and below	120	42.6
A level, ITE, Poly	73	25.9
University or above	89	31.6
Employment		
Unemployed	121	42.9
Employed	161	57.1
Marital Status		
Never Married	79	28.0
Ever Married	203	72.0
Relationship to care recipient		
Others	35	12.4
Son	48	17.0
Daughter	156	55.3
Spouse	43	15.2
Living with care recipient		
Yes	198	70.2
No	84	29.8
Have a domestic helper		
Yes	161	57.1
No	121	42.9
	Mean	S.D
Age	55.6	11.8
Average Weekly Caregiving Hours	54.9	52.9
Care recipient’s ADL	2.4	1.9
Care recipient’s IADL	5.9	1.9
Care recipient’s RMBPS	6.9	3.0
Social support	25.4	3.9
Outlook on life	12.0	2.5
Self-affirmation	23.3	4.7
DKAS factor 1	10.3	4.5
DKAS factor 2	7.8	3.4
DKAS factor 3	5.8	2.2

**Fig. 1:**
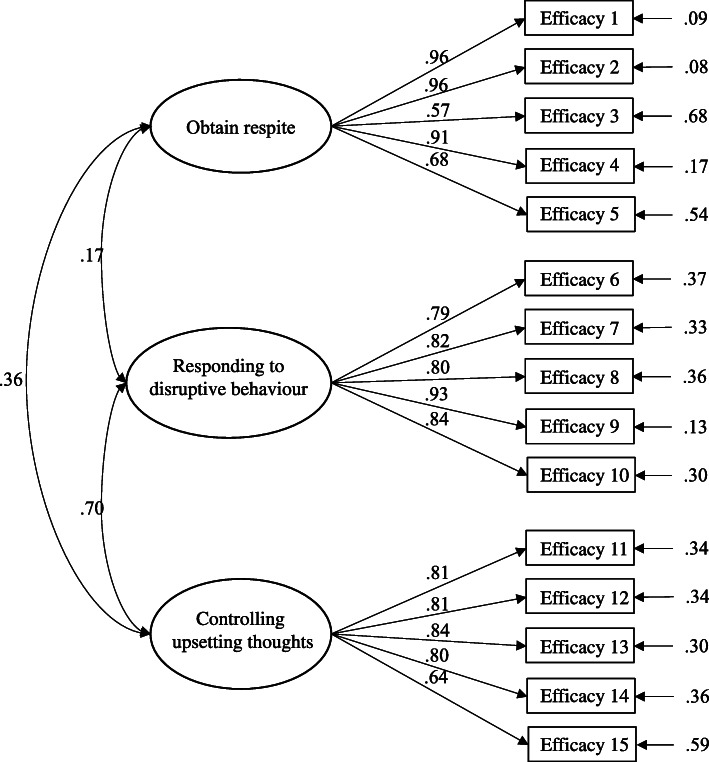
Figure of CFA path diagram

Our CFA of the RSCSE showed that the original proposed factor structure by the developer of the scale showed acceptable fit for our sample (i.e. CFI = 0.946, TLI = 0.935, and RMSEA = 0.086, slightly above 0.08 but still demonstrating mediocre fit [41]), and the standardized factor loading for all items varied from 0.570 to 0.959. The Cronbach’s coefficient for SE-OR, SE-RDB and SE-CUT factor were 0.906, 0.921 and 0.893 respectively. Refer to Table 2 for the descriptive statistics of items’ response and Fig. 1 for the path diagram representing the CFA of the RSCSE 3-factor model.

**Table 2:** Descriptive statistics of items’ response

Item	Frequency of NA responses	Frequency of Non-NA responses	Mean score of Non-NA responses
SE-OR			
How confident are you that you can ask a friend/family member to stay with care recipient for a day when you need to see your doctor?	3	279	74.3
How confident are you that you can ask a friend/family member to stay with care recipient for a day when you have errands to be done?	3	279	73.5
How confident are you that you can ask a friend/family member to do errands for you?	3	279	64.1
How confident are you that you can ask a friend/family member to stay with care recipient for a day when you feel the need for a break?	6	276	68.9
How confident are you that you can ask a friend/family member to stay with care recipient for a week when you need time for yourself?	7	275	46.7
SE-RDB			
When care recipient forgets your daily routine and asks when lunch is right after you’ve eaten, how confident are you that you can answer him/her without raising your voice?	8	274	71.2
When you get angry because care recipient repeats the same question over and over, how confident are you that you can say things to yourself that calm you down?	8	274	65.9
When care recipient complains to you about how you’re treating him/her, how confident are you that you can respond without arguing back?	20	262	64.2
When care recipient asks you 4 times in the first 1 h after lunch when lunch is, how confident are you that you can answer him/her without raising your voice?	15	267	63.4
When care recipient interrupts you for the fourth time while you’re making dinner, how confident are you that you can respond without raising your voice?	21	261	58.5
SE-CUT			
How confident are you that you can control thinking about unpleasant aspects of taking care of care recipient?	9	273	67.4
How confident are you that you can control thinking how unfair it is that you have to put up with this situation?	29	253	67.8
How confident are you that you can control thinking about what a good life you had before care recipient’s illness and how much you’ve lost?	36	246	69.3
How confident are you that you can control thinking about what you are missing or giving up because of care recipient?	34	248	71.5
How confident are you that you can control worrying about future problems that might come up with care recipient?	17	265	62.1

Multiple linear regressions found social support and Outlook on Life to be positively correlated to SE-OR. DKAS factor 1, Outlook on Life and average weekly caregiving hours were factors that were positively correlated to scores on SE-RDB. Caregivers who had a helper had significantly higher scores on SE-RDB, whereas caregivers who were living with the care recipient had significantly lower scores on SE-RDB. Lastly, age, social support and Outlook on Life were positively correlated to SE-CUT, while Self-Affirmation was negatively correlated to SE-CUT. Refer to Table 3 for the results of the multiple linear regressions.

**Table 3:** Multiple Linear Regression of the 3 factors of RSCSE

Factors	*SE-OR*			SE-RDB			SE-CUT		
	Β	95% CI	*p* value	β	95% CI	*p* value	β	95% CI	*p* value
Sex									
Male	−1.304	−15.29 to 12.68	0.854	−2.861	−14.99 to 9.268	*0.642*	7.627	−2.250 to 17.50	0.129
Female (ref)	**ref**	**ref**	**ref**	**ref**	**ref**	**ref**	**ref**	**ref**	**ref**
Ethnicity									
Indian and Others	−1.524	−14.27 to 11.22	0.814	−0.407	− 11.31 to 10.50	*0.941*	−7.682	−16.68 to 1.324	0.094
Malay	2.745	−8.470 to 13.96	0.630	−1.291	−10.88 to 8.302	*0.791*	4.052	−3.869 to 11.97	0.314
Chinese (ref)	**ref**	**ref**	**ref**	**ref**	**ref**	**ref**	**ref**	**ref**	**ref**
Education Level									
N/O level and below	0.437	−8.061 to 8.936	*0.919*	1.239	−6.041 to 8.519	*0.737*	−2.340	−8.344 to 3.664	0.443
A level, ITE, Poly	8.192	−0.333 to 16.71	0.059	3.640	−3.671 to 10.95	*0.327*	0.385	−5.636 to 6.407	*0.899*
University or above (ref)	**ref**	**ref**	**ref**	**ref**	**ref**	**ref**	**ref**	**ref**	**ref**
Marital Status									
Ever Married	−3.522	−11.13 to 4.090	*0.363*	−2.135	−8.713 to 4.442	*0.523*	3.981	−1.415 to 9.378	*0.147*
Never Married (ref)	**ref**	**ref**	**ref**	**ref**	**ref**	**ref**	**ref**	**ref**	**ref**
Employment Status									
Employed	3.738	−3.722 to 11.19	*0.324*	0.778	−5..601 to 7.158	*0.810*	1.431	−3.832 to 6.694	*0.592*
Unemployed (ref)	**ref**	**ref**	**ref**	**ref**	**ref**	**ref**	**ref**	**ref**	**ref**
Relationship to care recipient									
Son	1.342	−14.50 to 17.19	*0.867*	4.282	−9.478 to 18.04	*0.540*	−0.630	−11.80 to 10.53	*0.911*
Daughter	5.463	−7.847 to 18.77	*0.419*	−4.138	−15.52 to 7.246	*0.474*	4.437	−4.968 to 13.84	*0.353*
Others	4.730	−10.39 to 19.85	*0.538*	1.239	−11.71 to 14.19	*0.850*	4.973	−5.710 to 15.65	*0.360*
Spouse (ref)	**ref**	**ref**	**ref**	**ref**	**ref**	**ref**	**ref**	**ref**	**ref**
Living with care recipient									
Yes	−4.119	−12.30 to 4.069	*0.322*	−8.829	−15.86 to − 1.795	0.014*	−2.155	−7.933 to 3.623	*0.463*
No	**ref**	**ref**	**ref**	**ref**	**ref**	**ref**	**ref**	**ref**	**ref**
Domestic Helper									
Yes	6.358	−0.899 to 13.61	*0.085*	6.877	0.674 to 13.08	0.029*	3.342	−1.762 to 8.447	*0.198*
No	**ref**	**ref**	**ref**	**ref**	**ref**	**ref**	**ref**	**ref**	**ref**
Age	0.307	−0.060 to 0.675	*0.101*	0.201	−0.113 to 0.515	*0.209*	0.281	0.022 to 0.541	0.033*
Average weekly caregiving hours	−0.056	−0.131 to 0.018	*0.137*	0.065	0.001 to 0.128	0.045*	0.045	−0.007 to 0.098	*0.089*
ADL and IADL	−0.339	−1.327 to 0.648	*0.499*	−0.706	− 1.553 to 0.141	*0.102*	−0.216	− 0.911 to 0.478	*0.539*
RMBPS	−0.962	−2.058 to 0.132	*0.084*	−0.850	−1.799 to 0.098	*0.078*	−0.736	−1.508 to 0.034	*0.061*
Social Support	1.827	0.938 to 2.717	< 0.001**	0.005	0.103 to 1.537	*0.989*	0.940	0.311 to 1.568	0.003**
Self-Affirmation	−0.207	−1.029 to 0.614	*0.619*	0.155	−0.550 to 0.861	*0.664*	−0.798	−1.380 to − 0.217	0.007**
Outlook on Life	2.222	0.621 to 3.824	0.006**	2.728	1.357 to 4.100	< 0.001**	3.682	2.549 to 4.814	< 0.001**
DKAS factor 1	0.179	−0.657 to 1.016	*0.673*	0.820	0.103 to 1.537	0.025*	0.153	−0.437 to 0.745	*0.609*
DAKS factor 2	−0.481	−1.532 to 0.569	*0.367*	−0.828	− 1.727 to 0.071	*0.071*	−0.149	− 0.891 to 0.592	*0.691*
DKAS factor 3	−1.005	−2.566 to 0.554	*0.205*	−0.062	−1.395 to 1.269	*0.925*	−0.203	−1.300 to 0.894	*0.715*

DKAS factor 1: Misconceptions about dementia, DKAS factor 2: Caregiving considerations, and DKAS factor 3: Dementia Symptoms

*Denotes p value < 0.05, ** Denotes *p* value < 0.001

## Discussion

The sample of caregivers recruited in this study comprised mostly females (75.2%) with the majority being daughter caregivers (55.3%), and this is concordant to that of an earlier report published by Alzheimer’s Disease Association of Singapore, which revealed that 71.2% of caregivers in Singapore were females and majority of these caregivers were daughters (50.3%) [42]. The predominance of female caregivers in our sample is also consistent with the caregiving situation worldwide and further reinforces the notion that the typical dementia caregiver is likely to be female [1]. However, in contrast to Western populations where spousal caregivers are more common, daughters comprised the majority of our caregivers and this is likely due to the emphasis of filial piety in Asian societies [43]. The similarities of our sample’s characteristics with that of the report by Alzheimer’s Disease Association of Singapore -which aimed to profile the typical caregiver in Singapore- lends credence to our sample’s representativeness of the caregiving situation in Singapore.

Despite the cultural difference between the typical profile of a caregiver in Western and Asian population as described in the previous paragraph, our CFA of the RSCSE scale demonstrated that the factor structure proposed by the scale developer had an acceptable fit among informal caregivers in Singapore. The outcome of our CFA is also consistent with that of Cheng and colleagues (2013) [14] who employed a Chinese translated version of the scale and a sample of caregivers from Hong Kong, and that of Peñacoba and colleagues (2008) which utilized a Spanish translated version of the scale. The CFA of our study thus reinforces the robustness of the RSCSE’s 3-factor model across cultures.

An interesting finding which is worth mentioning is the significantly higher number of ‘not applicable’ responses to questions in the SE-CUT factor among our participants, which seems rather counterintuitive given that the questions in SE-CUT pertains to caregiver’s ability to control negative thoughts related to caregiving. We surmise that the 'not applicable' responses to items in SE-CUT could be attributed to Asian’s cultural values, insomuch that the expectations towards fulfilling familial duties (i.e. taking care of elder adults) and enacting filial obligations may have superseded their individual needs and desires [44, 45]. Hence, it is possible that the negative thoughts associated with caregiving described in SE-CUT were indeed ‘not applicable’ to the participants as such thoughts truly did not cross their mind. For instance, Wang (2012) cited in her review an example of a Taiwanese daughter whom people assumed would be angry towards her sisters for leaving her to care for her father alone, but was instead surprised at this comment when it was communicated to her because she had never thought that about her duty or about being angry [45]. Alternatively, it is also possible that the ‘not applicable’ responses by caregivers was due to social desirability bias or the feeling that it was inappropriate to convey negative feelings towards family, which was influenced by the of responsibility towards family in the Asian context [44].

Similar to the original study by Steffen et al. (2002) our study also found a positive association between perceived SE-OR and caregiver’s perceived social support. This positive relationship between SE-OR and perceived social support is easily explained, in that with greater social support, the caregiver is arguably more likely to be able to find someone to entrust their care recipient to when they feel the need for some respite. Social support was also significantly associated with SE-CUT. It has been posited that social support enhances an individual’s resilience to stress as it helps individuals reappraise the stressful situation that they are facing as well as to change their emotional reaction to it [46]. In which case, social support may help attenuate caregiver’s negative thoughts such as how life was better before they became a caregiver or how unpleasant their current caregiving situation is. In addition, caregivers with higher levels of social support are more likely to be able to obtain more respite, and this may help buffer against negative thoughts such as how unfair it is that they have to take on the role of a caregiver or having to miss out or give up certain things in life for the sake of caregiving.

Interestingly, having a domestic helper was not positively associated with SE-OR. We posit that this may be attributed to the way the questions were framed in the SE-OR factors in our study, in that we explicitly asked the respondents how confident they are in “finding a friend or family member” in assisting them for various scenarios. As a result, it is possible that some caregivers - whom did not consider their helper as a friend - may not have rated their confidence as highly in this aspect, even though they were still able to find some form of respite with help from their domestic helper. Hence, it is recommended that future studies looking to investigate caregiver’s SE-OR should consider reframing the questions to “seeking help from someone” rather than specifically friends and family. On the contrary, having a domestic helper was positively correlated with SE-RDB in this study. This is in line with the findings from another local study which found that having a domestic worker is associated with lower amount of caregiving provided by the caregiver, as well as reduced caregivers’ negative reaction towards caregiving duties [47]. It can be surmised that having a helper aids in the caregiver’s SE-RDB as caregivers are likely to have some form of assistance in dealing with caregiver’s behavioral issues. An earlier study by Tew et al. (2010) [22], found that caregivers without domestic helper are more likely to institutionalize their care recipient which also highlighted the importance of having a domestic helper. Locally, it may be helpful if the government could render assistance to the financially constrained caregiver, perhaps by further reducing or waiving the levy that comes with hiring a foreign domestic helper, or offering part-time dementia caregiving services.

In addition, caregivers with higher knowledge in DKAS of misconceptions about dementia (factor 1) [28] were associated with better SE-RDB. Presumably, caregivers who scored higher in DKAS factor 1 probably have less misperceptions about the condition, and are therefore able to respond to their care recipient’s problematic behavior more efficaciously, which in turns leads to a higher SE-RDB. While higher average weekly caregiving hours was positively associated with SE-RDB, living with care recipient was negatively associated with SE-RDB. Conceivably, caregivers with higher average weekly caregiving hours should have accumulated more experience on how to deal with or have become more inured to their care recipient’s disruptive behavior. On the other hand, caregivers who are living with the care recipient have a lower SE-RDB than those who are not. This was reported by Tew and colleagues (2010) that the dementia care recipient’s behavioral problems are one of the factors associated with institutionalizing of said care recipient. Taken together, such findings suggest the need to target and provide interventions for caregivers who are living with their care recipient on ways to handle their care recipient’s disruptive behaviors so as to improve their SE-RDB. Since better scores on DKAS factor 1 are linked to better SE-RDB, interventions for caregivers should also place an emphasis on imparting dementia knowledge or disabusing caregiver’s misperception towards dementia as it could affect their overall coping. An alternative for caregivers to improve their knowledge of dementia would be to utilize online learning resources, such as the “Understanding Dementia Massive Open Online Course”, which had demonstrated efficacy in improving knowledge of dementia for a diverse international learner group, regardless of educational background or prior experience with dementia [48].

Our analysis showed that higher scores on the Outlook on Life factor of the PAC scale was positively correlated to SE-OR, SE-RDB and SE-CUT. A model proposed by Kramer (1997) [49] suggests that caregiver’s self-efficacy and positive aspects of caregiving are associated insomuch that caregiver’s internal processes -such as their appraisal of stressors - are related to the caregiver’s ability to maintain a positive outlook throughout the caregiving process, and the results from this study reinforce this postulation. Alternatively, it is also possible that the relationship between Outlook on Life and the three constructs of self-efficacy measured by RSCSE have a bidirectional relationship, as it is posited that caregivers with higher self-efficacy would be more capable of identifying positive aspects of caregiving even in negative situations [50]. For instance, caregivers with a more positive Outlook on Life are more likely to reach out for help -a postulation that is corroborated by studies showing that a more positive outlook is linked to individuals adopting behaviors that are more beneficial to their health [51, 52] - thus enhancing their SE-OR. At the same time, when a caregiver is better able to obtain respite, the caregiver would have more time for their own interests and pursuits, which thus allows them to appreciate life more and thus improving their Outlook on Life. On the contrary, Self-Affirmation which is the other construct of the PAC scale, was found to be negatively associated with SE-CUT in our study. This particular finding appears to be counterintuitive given what was discussed above, and more studies should be conducted to further explore this phenomenon.

Lastly, our analysis also revealed a positive association between age and SE-CUT. This could be due to the “positivity effect” phenomenon, an age-related trend whereby relative to the younger counterparts, older people remember and pay attention to more positive than negative stimuli [53]. In which case, older caregivers might be able to control upsetting thoughts about caregiving better because they potentially ruminate less on the negative aspects. On the other hand, it is also possible that the relationship between age and SE-CUT is due to other factors such as the stage of life the caregiver is in, because arguably, a younger individual at the threshold of his or her career who takes up the role of a caregiver has more sacrifices to deal with (i.e., hindering the progression of their career because of the need to devote time and attention to caregiving) as compared to one who is retired.

Findings from this study provided insights into the factors that influence informal caregivers’ self-efficacy in caregiving. Crucially, since a better Outlook on Life is linked to greater self-efficacy in all domains, this signals the need to help caregivers adopt a more positive outlook in order to bolster their caregiving efficacy. A viable strategy to achieve this would be to introduce interventions that aims at imparting cognitive restructuring skills for informal caregivers, and this could be achieved perhaps through cognitive behavioral therapy, which has also been reported to significantly improved the depressive symptoms of caregivers of PWD [54].

In addition, this study also highlights the importance of social support for informal caregiver’s self-efficacy. Hence, it is recommended for the informal caregivers in Singapore to broaden their support network, and one way to accomplish this would be to join caregiver support groups considering that caregiver support interventions has been found to confer positive effects on caregiver’s coping ability, knowledge and social support [55]. In Singapore, such support groups tailored for dementia caregivers are usually free to join and are run by various organizations. For instance, the Alzheimer’s Disease Association (ADA) in Singapore offers a 2-h support session twice a month, which consists of a talk by a guest speaker followed by a sharing session amongst caregivers. Besides the ADA, there are also some hospitals in Singapore which offer free support group sessions for caregivers such as the Khoo Teck Puat Hospital (KTPH), which runs a dementia support group that is open to all caregivers, and also provides meetup sessions for caregivers to interact with the KTPH dementia care team to discuss about issues related to dementia and caregiving. As another option, caregivers could also consider joining online peer support groups, which have been reported to alleviate caregiver’s stress [56]. This approach also allows caregivers to get more real-time support, maintain a greater degree of privacy, and obviates the need for caregivers to travel to a physical location which may be particularly beneficial for the busier caregivers.

### Limitations

A notable weakness in this study is that we did not manage to collect care recipient’s clinical diagnosis, hence we were not able to determine whether the stages and type of dementia affect caregiving self-efficacy. Nonetheless, we collected care recipients’ RMBPS, ADL and IADL scores which we believe provided a good enough reference for how patient’s symptoms affect caregiving efficacy. However, we were unable to collect care recipients’ sociodemographic information such as sex and age, and were thus unable to determine if such factors would influence caregiver’s self-efficacy. As such, it is recommended for future similar studies to collect patient’s sociodemographic information as well. Also, the study sample was recruited via convenience sampling and participants were self-selected, and therefore the results from this study may not be generalizable to all informal caregivers of PWD in Singapore. Another limitation to highlight is that the RMSEA value of our CFA though still acceptable [41], was slightly higher than the recommended cut-off of 0.08. In this case, it may be worthwhile for future studies to further examine the stability of this 3-factor model among a bigger sample of informal caregivers. Lastly, social desirability bias may also have influenced the results. There is a possibility that participants may have rated their confidence higher than what is actually reflective of their true confidence for the questions loading on the SE-RDB and SE-CUT subscales.

Notwithstanding these limitations, this study elucidated important novel insights into the factors that influences caregiving self-efficacy amongst informal caregivers in Singapore, which could help inform the design of interventions and policies that could benefit these individuals in future.

## Conclusion

The extant literature has evinced that having a higher self-efficacy is linked to better health and coping outcomes for the informal caregivers of PWD. To our knowledge, this is the first study that examined the correlates of the multiple domains of caregiving self-efficacy among informal caregivers in Singapore. The proposed 3-factor structure by the scale originator [12] has an acceptable fit in our sample. The findings of this study also highlighted the importance of having social support and a positive outlook on life for caregiver’s self-efficacy, as well as the recommendations of possible interventions to improve their self-efficacy. Finally, future similar studies could be replicated in non-self-selected sample of caregivers, or employ a self-administered approach to survey participants to investigate the generalizability of our findings.

## Data Availability

The datasets used and/or analysed during the current study are available from the senior author at mythily@imh.com.sg on reasonable request.

## Abbreviations

PWD: Persons with dementia
CFA: Confirmatory factor analysis
RSCSE: Revised scale for caregiving self-efficacy
ADL: Activities of daily living scale
IADL: Instrumental activities of daily living scale
RMBPC: Revised memory and behavioral problems checklist
PAC: Positive aspects of caregiving scale
DKAS: Dementia knowledge assessment scale
SE-OR: Self-efficacy in obtaining respite
SE-RDB: Self-efficacy in responding to disruptive behaviors
SE-CUT: Self-efficacy in controlling upsetting thoughts
CFI: The comparative fit index
TLI: Tucker-Lewis index
RMSEA: Root mean square error of approximation
